# Haptic technology in orthodontics: a systematic review and meta-analysis

**DOI:** 10.3389/froh.2026.1768079

**Published:** 2026-06-09

**Authors:** Mohammad Khursheed Alam, Suliman Alsaeed, Mohammad Younis Hajeer, Huda Abutayyem, Afnan Alayyash

**Affiliations:** 1 Preventive Dentistry Department, College of Dentistry, Jouf University, Sakaka, Saudi Arabia; 2 Department of Dental Research Cell, Saveetha Institute of Medical and Technical Sciences, Saveetha Dental College and Hospitals, Chennai, India; 3 Department of Public Health, Faculty of Allied Health Sciences, Daffodil International University, Dhaka, Bangladesh; 4 Preventive Dental Sciences Department, College of Dentistry, King Saud bin Abdulaziz University for Health Sciences, Riyadh, Saudi Arabia.; 5 King Abdullah International Medical Research Center, Riyadh, Saudi Arabia; 6 Ministry of the National Guard – Health Affairs, Riyadh, Saudi Arabia; 7 Department of Orthodontics, Faculty of Dentistry, University of Damascus, Damascus, Syria; 8 Department of Clinical Sciences, Center of Medical and Bio-Allied Health Sciences Research, College of Dentistry, Ajman University, Ajman, United Arab Emirates; 9 Preventive Dentistry Department, Taif University, Taif, Saudi Arabia.

**Keywords:** bracket bonding, haptic technology, orthodontic education, orthognathic surgical planning, simulation-based training, virtual reality

## Abstract

Tactile feedback through haptic technology is increasingly being applied in orthodontic education and selected clinical workflows, but its overall effectiveness has not been comprehensively synthesized. This systematic review and meta-analysis aimed to summarize the evidence on haptic and virtual reality technologies in orthodontic training and practice. In accordance with the Preferred Reporting Items for Systematic reviews and Meta-Analyses guidelines, a systematic search of multiple databases was conducted from inception through October 2025. Nine studies met the Population, Intervention, Comparison, and Outcome-based eligibility criteria. A random-effects meta-analysis was performed to estimate effect sizes (Hedges’ g), and subgroup analyses were undertaken to explore heterogeneity. The pooled effect size was large and statistically significant (g = 1.40, 95% CI: 0.82–1.98, *p* < 0.001), although heterogeneity was high (I^2^ = 77.32). A subgroup analysis indicated beneficial effects in both bracket-bonding training and orthognathic surgical planning, with greater variability in bracket-bonding studies. Although the evidence base is limited by moderate risk of bias in several studies and a small number of trials, haptic and VR systems appear valuable for improving psychomotor learning and procedural planning in orthodontics. Their educational efficacy is supported, whereas translation to direct patient-care benefits remains promising but not yet firmly established.

**Systematic review registration:**

https://www.crd.york.ac.uk/prospero/display_record.php?ID=CRD420251246154, PROSPERO 2025 CRD420251246154.

## Introduction and background

Haptic technology has been revolutionary in the field of dentistry and orthodontics education and practice, with a significant impact on precision, skills, and treatment results. As it is a specialty that revolves around the correction of malocclusion and dental abnormalities, the adoption of new digital technologies, such as three-dimensional (3D) imaging, 3D printing, and other haptic simulators, positively influences the accuracy of diagnosis and treatment planning because practitioners can now see and manipulate complex anatomy in 3D (1, 2).

The tactile sensations generated by the use of haptic devices in a procedure are essential in developing psychomotor skills in a risk-free set-up where learners can be confident and competent in their work before they handle patients (1, 3). Recent technological innovations involve the use of mixed reality (MR) and virtual reality (VR) simulators that integrate both visual and force feedback to produce immersive clinical settings, which can increase learner engagement. hand- eye coordination, and the transfer of skills to real-life settings (3–5).

In orthodontics, the main documented applications of haptic and immersive technologies to date are educational simulation, bracket-placement training, and orthognathic planning rather than routine patient-facing force-monitoring systems. This distinction is important because the strongest current evidence relates to skill acquisition, spatial understanding, and procedural rehearsal in controlled training environments (1, 3).

Although there are some encouraging signs, there is a low uptake of haptic technology in orthodontics. A survey of dental schools in the Gulf Cooperation Council countries found that an insignificant proportion (6.7%) currently use haptic virtual reality simulators in their academic programs, yet almost half are interested in acquiring this technology in the future (3). The obstacles to extensive adoption encompass high financial costs, the small number of patient cases in simulator libraries, and the absence of standardized protocols for clinical and educational applications (2, 3). Furthermore, faculty distrust and insufficient curricular incorporation are obstacles to exploiting artificial intelligence (AI) and haptic technology in orthodontic education to the fullest extent (2). To overcome these obstacles, it is necessary to implement strategic curricular changes, focused faculty development, and additional research to establish the clinical efficacy and cost-efficacy of these technologies.

At present, the literature more consistently supports educational and planning benefits than direct improvements in patient-level clinical outcomes. Accordingly, claims regarding reduced treatment time, fewer bond failures, or better patient outcomes should be interpreted cautiously unless they are directly linked to haptic or immersive orthodontic interventions evaluated in comparative studies (4).

Overall, haptic technology in orthodontics is a developing field with considerable potential, but the evidence base remains narrow and methodologically heterogeneous. A focused synthesis is therefore needed to clarify where benefits are already supported, where translation to clinical practice remains hypothetical, and what gaps should guide future research.

## Review

### Methodology

This meta-analysis and systematic review was conducted in accordance with the Preferred Reporting Items for Systematic reviews and Meta-Analyses (PRISMA) guidelines (6) to identify, select, and synthesize studies on haptic technology in orthodontics. Several electronic databases were searched with the help of pre-determined keywords and Medical Subject Headings (MeSH) terms connected with orthodontics and haptic technology. The inclusion criteria were studies that assessed the clinical use of haptic technology and educational devices or diagnostic equipment involving haptic feedback.

### Search strategy rationale and implementation

A general search strategy was used in various databases, including PubMed, Scopus, Web of Science, Embase, and Cochrane Library, to collate a wide range of studies on the use of haptic technology in orthodontics. Boolean operators were used to combine key concepts so that relevant studies on orthodontic applications and haptic feedback technologies were included. To improve the relevance and quality of the results, filters were used to restrict the results to human studies published in English over the past 10 years and clinical trials and observational studies. MeSH and Emtree terms, phrase searching, truncation, and proximity operators, which are database-specific syntax, were used to maximize search sensitivity and specificity. This organized approach maximized the chances of retrieving relevant articles and minimized the number of irrelevant records, leading to the creation of a strong evidence synthesis (Table 1).

**Table 1:** Search strategy in the databases for the systematic review of haptic technology in orthodontics.

Database	Search query components	Applied filter	Syntax/modifier
PubMed, Scopus, and Web of Science	(“Orthodontics” OR “Dental Orthodontics”) AND (“Haptic Technology” OR “Force Feedback” OR “Dental Simulation”)	English language, human studies, and in the previous 10 years	Boolean operators (AND, OR), phrase searching with quotes, and truncation (*)
Embase	(“orthodontics”/exp OR orthodontics) AND (“haptic technology” OR “force feedback” OR ”dental simulation”)	English language, human studies, and clinical trials and reviews	Emtree terms, Boolean operators, and proximity operators
Cochrane Library	(“Orthodontics” AND “Haptic Technology”)	Systematic reviews and clinical trials	Phrase searching and Boolean operators

* denotes truncation/wildcard searching used to capture word variants in database search syntax.

Besides the search of the electronic databases, a manual search was also performed by screening the reference lists of the included articles and relevant reviews to acquire studies that may have eluded the automatic searches. Grey literature and conference proceedings were also reviewed in order to limit bias in publications. Study selection and data extraction were carried out by two independent reviewers, with any disagreements settled by consensus discussion. In case of disagreement, a third reviewer was approached in order to make unbiased decisions. The result of this rigorous process is that the reliability and validity of the review findings are improved due to the minimal presence of selection bias and full coverage of the literature.

### Rationale for eligibility criteria

The Population, Intervention, Comparison, and Outcome (PICO) framework (7) was used to define the eligibility criteria for this review. The population comprised patients undergoing orthodontic treatment or trainees in orthodontic education. Eligible interventions were haptic technologies or simulators specifically applied to orthodontic procedures or orthodontic training. Comparators included conventional orthodontic methods or the absence of haptic feedback. Outcomes of interest included clinical or procedural measures, such as treatment accuracy and force-related performance, and educational outcomes, such as skill acquisition and user satisfaction. Only randomized studies, controlled clinical studies, and observational studies published from 2010 through October 2025 were included; case reports, editorials, and opinion pieces were excluded. This framework enabled a clear and structured study-selection process that was aligned with the objectives of the review (Table 2).

**Table 2:** Eligibility criteria for the meta-analysis using the PICO framework (7).

PICO component	Inclusion criteria	Exclusion criteria
Population	Patients undergoing orthodontic treatment or trainees in orthodontic education	Non-human studies and unrelated to dental specialties
Intervention	Use of haptic technology devices or simulators in orthodontic procedures or training	Studies without haptic feedback or simulation
Comparison	Conventional orthodontic methods or no haptic technology	Studies lacking a comparison group
Outcomes	Clinical outcomes (treatment accuracy and force measurement) or educational outcomes (skill acquisition and user satisfaction)	Studies without measurable outcomes or qualitative only
Study design	Randomized controlled trials, controlled clinical trials, and observational studies	Case reports, expert opinions, and editorials

### Data extraction process

Two reviewers used a standardized form to extract data using their own interpretation of the key characteristics of the studies (author details, year of publication, study design, population demographics, details of interventions, comparison groups, outcome measures, and results) in two independent reviews. Extracted data were cross-checked to ensure that they were accurate and complete. The authors were contacted if there was any missing information. The systematic methodology meant that the collection of data was uniform and thorough, which allowed for the synthesis and meta-analysis of evidence regarding the use of haptic technology in orthodontics.

### Quality and bias assessment

The Revised Cochrane risk-of-bias tool for randomized trials (RoB 2) was used to evaluate the risk of bias in the randomized controlled trials (8), assessing the level of bias in the following domains: randomization, deviation of intended interventions, absence of outcome data, outcome measurement, and selective reporting. The risk of bias in non-randomized follow-up studies of exposure effects (ROBINS-E) tool (9) was used to evaluate the risk of bias in the non-randomized studies in the following domains: confounding, selection bias, classification of interventions, and outcome measurement. Visual inspections of funnel plots and Egger’s regression test were used to provide evidence of asymmetry and publication bias (10). These evaluations were a critical analysis of the studies’ validity and how bias could affect the meta-analysis findings.

### Statistical analysis approach

Quantitative data were pooled using random-effects meta-analysis models. The effect sizes were computed as standardized mean differences or risk ratios with a 95% confidence interval based on the type of outcome. The I^2^ statistic and Cochran’s Q test were used to determine heterogeneity. Subgroup analyses were conducted among the orthodontic studies to investigate the variation in pain based on treatment type (fixed appliance vs. aligners), haptic technology type MR vs. VR), and/or treatment phase (e.g., leveling/alignment vs. finishing), and anatomical orientation (upper vs. lower arch) that could be reduced through haptic technology application. Finally, sensitivity analyses were performed. A *p*-value  < 0.05 was determined to be significant. Proper software was utilized in all the analyses in order to generate accurate and reproducible results. The synthesis of the evidence was rigorous and reproducible as all the reviews were performed on the proven statistical software Review Manager (RevMan), version 5.4 (11).

## Results

### Study-selection process

**Figure 1:**
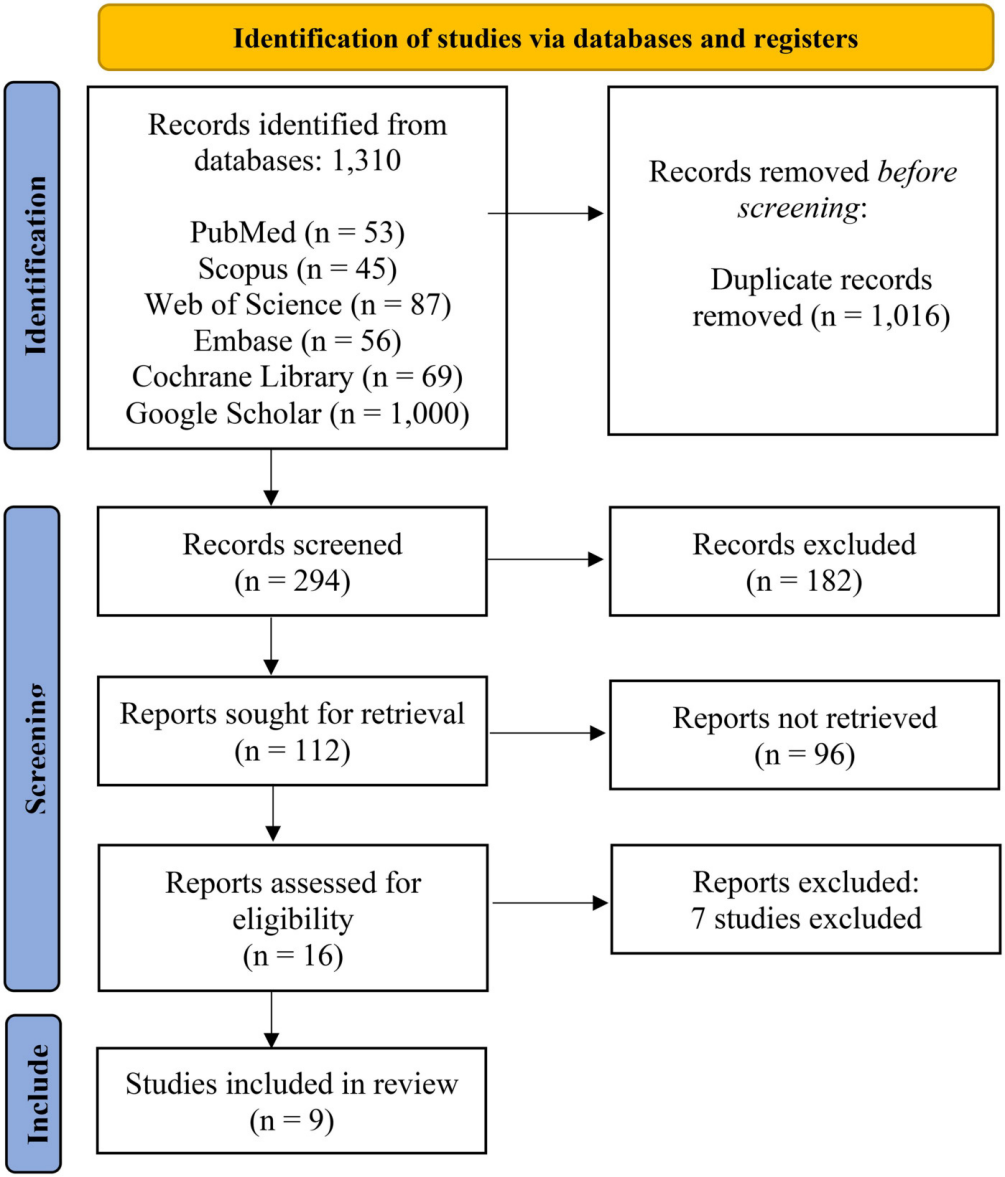
Literature search flowchart.

We searched six major electronic databases and initially identified 1,310 records. After the removal of 1,016 duplicate records, 294 unique records underwent title and abstract screening. Of these, 112 records were sought for retrieval, 96 were not retrieved, and 16 full-text reports were assessed for eligibility. Seven studies were excluded for prespecified reasons (12–18) (Table 3), leaving nine studies for qualitative synthesis and meta-analysis (19–27). The PRISMA flowchart summarizing the study-selection process is presented in Figure 1.

**Table 3:** Examples of excluded studies and rationale for exclusion.

Reference	Citation	Primary reason for exclusion
(12)	Li et al. (2025)	Irrelevant intervention and population: Focuses on VR for veneer preparation in restorative dentistry, not orthodontics. The study does not specify the use of haptic technology
(13)	Hu et al. (2025)	Irrelevant population and intervention: Pertains to endodontic (root canal) training, not orthodontics. The simulation focus is not specified as haptic
(14)	Sheng et al. (2022)	Irrelevant intervention and population: Concerns inlay preparation in restorative dentistry. The “jaw simulation” likely refers to a physical model, not a haptic virtual one
(15)	Im et al. (2025)	Irrelevant intervention: The focus is on training for dental radiography, which is a diagnostic imaging procedure, not a tactile orthodontic procedure
(16)	Pulijala et al. (2018)	Irrelevant population: Focuses on oral and maxillofacial surgery training (e.g., mandibular fracture), which is a surgical specialty distinct from orthodontics
(17)	Eom et al. (2025)	Irrelevant intervention and outcomes: The intervention is aimed at improving ergonomics and posture, not simulating orthodontic procedures or providing haptic feedback for skill acquisition
(18)	Almugait et al. (2021)	Irrelevant intervention and outcomes: Uses VR purely as a distraction tool for patient anxiety during anesthesia, not as a haptic simulator for clinician training or procedure execution

The synthesized evidence in this review from the included nine studies reveals that haptic and virtual reality technologies are being actively researched and implemented in many aspects of the field of orthodontics, mainly in the area of education and surgical design. The study designs included in the research varied, including randomized controlled trials, longitudinal studies, and observational studies. One of the recurring results, found in several studies, is that these technologies do improve the learning process, resulting in better practical skills in terms of bracket bonding accuracy and surgical planning proficiency among students and trainees. In addition, there were promising signs that these tools can fill the experience gap, as less-experienced clinicians demonstrated comparable precision when using the haptic or augmented guidance systems. The outcome measures included various aspects, i.e., the acquisition of skills, accuracy, and efficiency, user confidence, and satisfaction, and the majority of the studies showed positive outcomes, promoting the introduction of these technologies into orthodontic education and practice (Table 4).

**Table 4:** Summary of included studies on haptic technology in orthodontics.

Author(s), year	Study design	Population	Intervention (haptic technology)	Comparison	Outcome measures	Key results/conclusions
Liu et al., 2025 (19)	Longitudinal study	First-year orthodontic postgraduate students (*n* = 26)	Haptic-enhanced VR simulator for orthodontic bracket bonding training	Traditional training (phantom head with typodont)	Bonding accuracy, performance scores, and operation time	The virtual training system significantly boosted students’ bracket bonding scores by approximately 50% (*p* < 0.05), with 88.46% of students agreeing it provided a realistic clinical simulation
Liu and Bin, 2025 (20)	Longitudinal study	Twenty-six orthodontic postgraduates (6 men, 20 women)	Haptic-enhanced VR simulator for orthodontic bracket bonding training	Self-comparison over time	Bonding accuracy, performance scores, and operation time	The virtual training system significantly increased bracket bonding scores by approximately 50% (*p* < 0.05), with 88.46% of students attributing their improvement to its realistic simulation and real-time feedback. Progressive practice results across multiple skill items also showed statistically significant gains of 3%–17% (*p* < 0.05)
Ströbele et al., 2025 (21)	Observational study	Experienced vs. inexperienced dentists (*n* = 5, each)	Augmented reality (AR) system with haptic cues for bracket placement	Performance compared between experienced and inexperienced users	Bracket placement accuracy (linear and angular deviations)	The inexperienced dentists were more precise in bracket positioning than the experienced dentists without AR usage. However, AR improves the positioning in dentists rather than orthodontists. The significance value was (*p* < 0.004)
Li et al., 2021 (22)	Observational study	Thirty four participants, divided into a novice group (*n* = 17) and a skilled group (*n* = 17)	Mixed reality and haptic-based dental simulator for tooth preparation	Preliminary evaluation	System usability, realism, and performance	A strong majority (74%) of the participants were satisfied with the Unidental MR simulator, with over 90% agreeing it stimulated learning interest. While the majority of the user experience metrics showed no significant difference between study groups, overall satisfaction was positively correlated with the headset's resolution (*p* = 0.03) and the realism of the preparation process (*p* = 0.001)
Sytek et al., 2021 (23)	Observational study	Twenty graduate orthodontic residents	Virtual reality (VR) surgical simulation for orthognathic treatment planning	2D prediction tracing and 3D simulation	Diagnostic quality, treatment planning confidence, and user attitudes	The 3D and VR surgical simulations led to a significantly greater number of prescribed surgical movements and longer task completion times compared to other methods (*p* < 0.001)
Sakowitz et al., 2020 (24)	Randomized controlled trial	Thirty third-year dental students	Virtual reality patient (VRP) for orthognathic diagnosis/treatment planning	2D prediction tracing	Diagnostic and treatment planning accuracy and efficiency	The educational intervention significantly improved students’ multiple-choice question (MCQ) exam scores (*p* < 0.05), and both 2D and VR methods enhanced their understanding of case planning. However, no significant difference was found between the two groups’ performance on written case analyses or across all MCQ test periods, despite scoring reliability being high (>0.928)
Wan et al., 2024 (25)	Randomized controlled trial	Twenty fifth-year medical students	Immersive virtual reality (IVR) surgical training system for orthognathic surgery	Traditional textbook-based learning	Theoretical knowledge, practical skill scores, and confidence	The VR group achieved higher scores than the traditional group (94.67 vs. 87.65). Compared with the control group, the VR group completed the procedure more quickly, with fewer instrument selection and angular errors
Stevanie et al., 2025 (26)	Randomized controlled trial	Twelveparticipants, including surgery experts, IR specialists, engineering students, and residents	Immersive reality (IR) for Le Fort I orthognathic surgery training	Conventional training (lectures, videos, CT analysis)	Procedural understanding, task completion, and user satisfaction	Based on the evaluation using the IRNQ questionnaire, the IR technology received high average scores across all key parameters, with In-App Assistance (30.93) and IRISE (32.46) rated most favorably. The Game Mechanism (28.75) and User Experience (30.72) parameters also demonstrated strong positive reception
Chen et al., 2025 (27)	Observational study	Fifty undergraduate dental students	Virtual reality (VR) system for orthodontic education (bracket bonding, wire bending)	N/A (evaluation of the system's implementation)	Learning satisfaction, skill acquisition, and system usability	The VR group demonstrated higher learning motivation and a more positive learning experience than the PPT group, with a clear majority recommending VR over traditional methods. However, the overall differences between the two groups were generally small, and some specific comparisons showed no significant advantage for VR

2D, two-dimensional; 3D, three-dimensional; AR, augmented reality; CT, computed tomography; IVR, immersive virtual reality; IR, immersive reality; PPT, PowerPoint presentation; RCT, randomized controlled trial; VR, virtual reality; VRP, virtual reality patient.

There is plentiful evidence underpinning the application of haptics-enhanced simulators in the acquisition of skills in orthodontic education. Liu et al. (19) conducted two longitudinal studies that were based on the hypothesis that dental students who practiced bracket bonding using a haptic VR simulator had much higher accuracy and performance scores and lower operation times as opposed to the group trained on standard phantom heads (19, 20). This means that VR training can be not only effective but also more efficient.

Equally, Chen et al. (27) observed that VR-based orthodontic bracket bonding and wire bending training was perceived as very satisfactory and effective among undergraduate students for developing their skills. This technology has also proven useful in complicated surgical operations (27).

In an RCT conducted by Wan et al. (25), medical students who received orthognathic surgery training using an immersive virtual reality (IVR) system demonstrated a much higher level of improvement in theoretical knowledge, practical skills, and self-confidence in comparison with a control group that trained using traditional textbook learning (25). Stevanie et al. (26) confirmed this in an RCT where an immersive reality (IR) simulator of Le Fort I osteotomy provided to surgical trainees led to the trainees gaining superior knowledge of the surgical procedure and having enhanced satisfaction (26).

Haptic and VR technologies also demonstrate great advantages in the diagnostic and planning stages of orthodontic and surgical treatment. In an RCT, Sakowitz et al. (24) found that dental students in the virtual reality patient (VRP) group, i.e., those who applied the VRP module to orthognathic planning, produced far fewer errors when making diagnoses and treatment plans and performed the tasks in a shorter time compared to the students in the control group, i.e., those who used conventional two-dimensional (2D) prediction tracing (24).

These findings were supported by an observational study conducted by Sytek et al. (23), which revealed that, when using VR surgical simulation to plan the case, orthodontic residents scored higher in the quality of their diagnosis and confidence with their choice of treatment plan than when using 2D or 3D planning (23).

The use of this technology as an aid tool in clinical processes is an advantage. In an observational study by Ströbele et al. (21), when bracket placement was performed with the help of an augmented reality (AR) system with haptic cues, inexperienced dentists reached a comparable level of placement accuracy as experienced clinicians (21), which indicates the possibility of standardizing outcomes and minimizing errors.

Li et al. (22) focused on a tooth preparation simulator. Their initial assessment proved that the system is useful and capable of distinguishing between people with varying skill levels, which indicates the possibility of using haptic feedback for training motor skills in dentistry (22).

### Comparative risk of bias assessment in randomized and non-randomized clinical studies

#### Risk of bias

**Figure 2:**
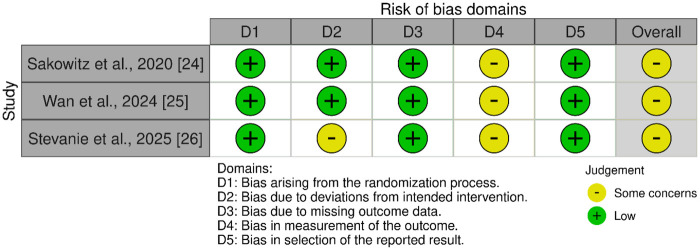
Risk of bias assessment of the randomized controlled trials (RoB 2) (8).

The three RCTs were generally evaluated as having “some concerns.” Incorrect measurement of the outcome was the main source of bias in these studies, i.e., domain 4 (D4) in the RoB 2 tool. This indicates that there was a possibility that the means of the above measurements (e.g., knowledge scores, practical skills, and user satisfaction) were affected by the knowledge of the participants or the assessors of which intervention they received. In other examples, such as those assessing educational tools, the participants who were aware of being in the innovative VR group may have had increased self-reported confidence or motivation. Furthermore, regarding Stevanie et al. (26), there were “some concerns” in domain 2 (D2), namely, deviation from the planned intervention that may have influenced the results (Figure 2).

**Figure 3:**
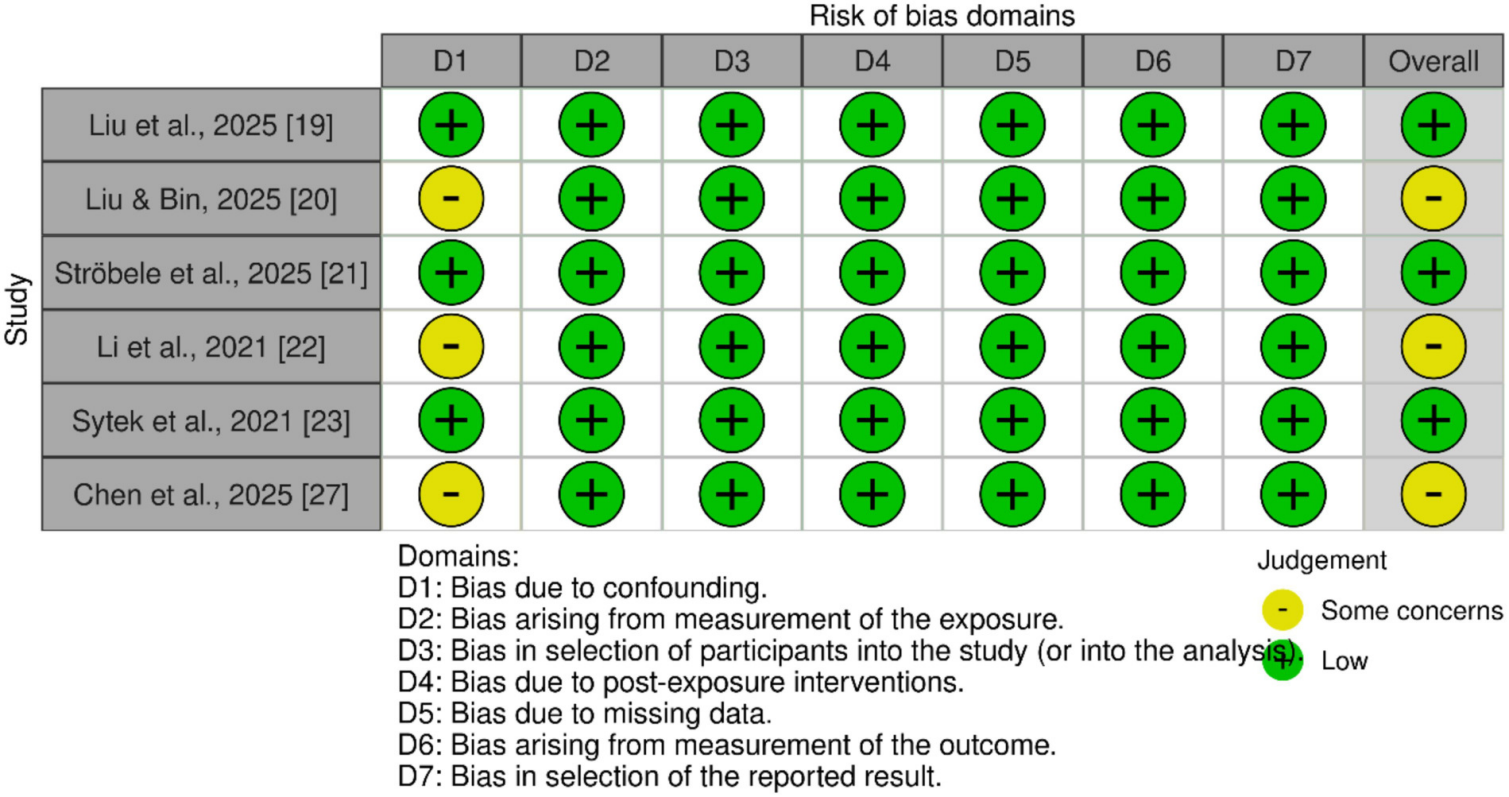
Risk of bias assessment of the non-randomized studies (ROBINS-E) (9).

The evaluation demonstrated a distinct division of quality. Three studies (19, 21, 23) were deemed to have a “low” overall risk of bias and had good performance in all domains. The remaining three studies (20, 22, 27) were considered to have “some concerns” regarding their risk of bias. The overall weakness of these three was in domain 1 (D1), i.e., bias due to confounding. This is a widespread and significant drawback of single-arm research or studies that lack a strong comparator cohort, as they cannot control for issues such as previous student capabilities, the development of the learning process, or motivation, which are likely the actual reasons for the observed results, not the intervention itself (Figure 3).

#### Publication bias

**Figure 4:**
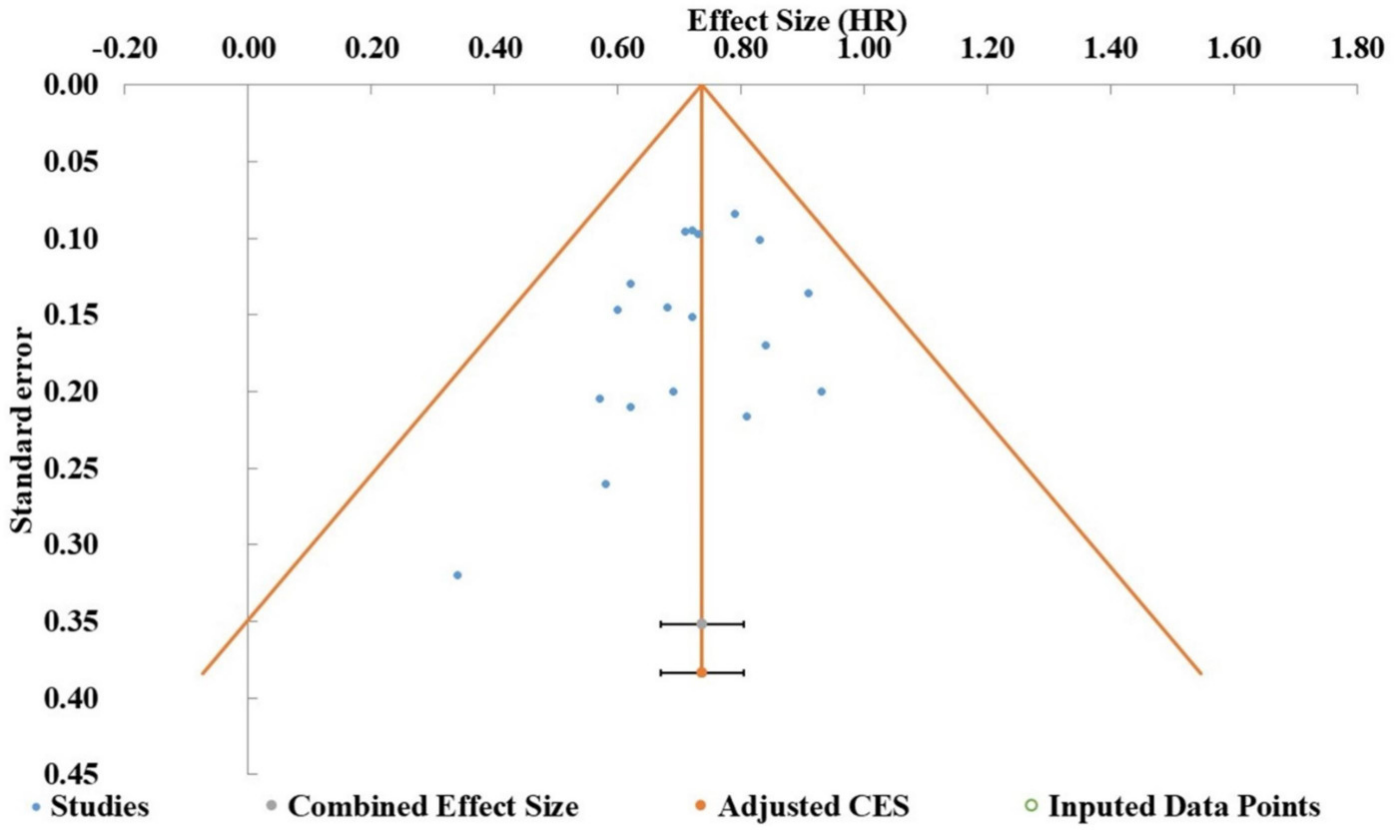
Publication bias funnel plot.

The funnel plot illustrates that the distribution of each study’s effect size (Hedges’ g) around the combined effect size is generally asymmetric, with a few smaller studies (with higher standard error) with large positive effects concentrated at the top and a significant absence of smaller studies with negative or no effect on the left side of the plot. This asymmetry implies a possible bias in which smaller studies that have significant, positive, results are more likely to be published and smaller studies with non-significant or negative results are absent from the literature. The Egger’s test results provided a statistical assessment of the asymmetry of the funnel plot (Table 5). The most important aspect is the non-significant *p*-value, i.e., 0.108, of the intercept. The Egger’s test results show that although the funnel plot is visually suggestive, the asymmetry is not statistically significant. The positive intercept (3.86) is an indication of the possibility of larger effects in the smaller studies; however, the broad confidence range between −0.98 and 8.70 indicates the uncertainty of such an estimate (28, 29). The publication bias funnel plot is shown in Figure 4.

**Table 5:** Egger's regression test for funnel plot asymmetry (10).

Parameter	Estimate	Std. error	95% CI lower limit	95% CI upper limit
Intercept	3.86	2.10	−0.98	8.70
Slope	−0.14	0.77	−1.90	1.63
t-value	1.84			
*p*-value	0.108			

## Meta-analysis findings

### Forest plot

**Figure 5:**
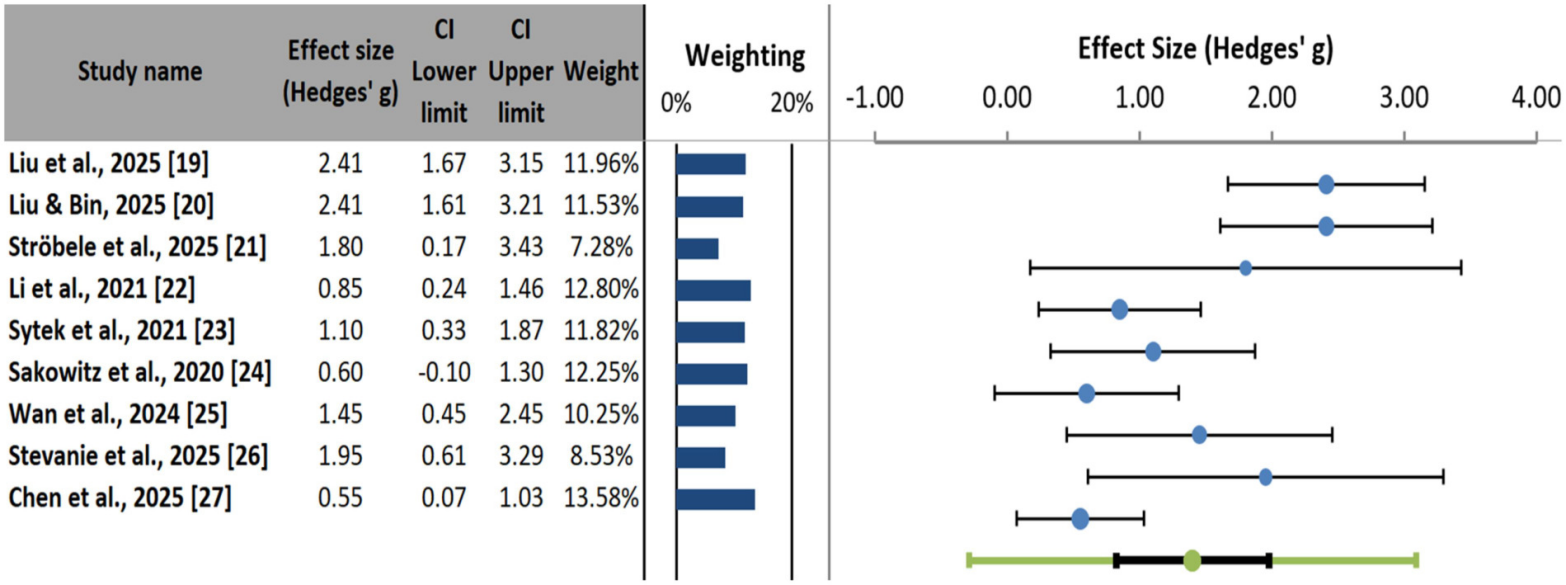
Forest plot of the individual study and pooled effect sizes.

The forest plot visually summarizes the results of the meta-analysis and shows the effect size (Hedges’ g) and the 95% confidence interval of each of the nine studies incorporated in the meta-analysis, along with the measures of the overall pooled effect. The individual study effect sizes are all positive, which means that haptic/VR technology had a beneficial effect in all the studies. The majority of the studies had a statistically significant effect, as the confidence intervals did not intersect the no effect line (g = 0). It is important to note that the studies of Liu et al. (19) and Liu and Bin (20) showed the greatest effects (Figure 5).

### Heterogeneity assessment

The random effects meta-analysis demonstrated that there was a large statistically significant positive effect of the use of haptic and virtual reality technology in orthodontics, with a pooled Hedges’ g of 1.40 (95% CI: 0.82–1.98). The statistical significance of this result was proven by a *p*-value of less than 0.001. Nonetheless, this analysis showed that there was high heterogeneity among the studies, as indicated by a highly significant Cochran’s Q test result (*p* less than 0.001) and a high I^2^ value of 77.32. This indicates that approximately 77% of the variation in the observed effect can be explained by real differences across the studies and not sampling error. The prediction interval, i.e., the range within which the actual effect of a future study is estimated to be, is between −0.29 and 3.09, indicating that the overall effect may be positive, but the effect in a new environment may be either insignificant or even more significant. This heterogeneity highlights the differences among the included studies with regard to the type of technology, outcome measures, and population, which justifies the application of a random effects model (30) (Table 6).

**Table 6:** Summary of the random effects meta-analysis and heterogeneity.

Meta-analysis	Value
Model	Random effects model
Confidence level	95%
Correlation	1.40
Effect size (correlation)	0.25
Confidence interval, lower limit	0.82
Confidence interval, upper limit	1.98
Prediction interval, lower limit	−0.29
Prediction interval, upper limit	3.09
Z-value	5.58
One-tailed *p*-value	0.000
Two-tailed *p*-value	0.000
Number of included studies	9
Heterogeneity statistics	
Q (Cochran's)	35.28
pQ	0.000
I^2^	77.32%
T^2^ (tau-squared)	0.47
T (tau)	0.69

### Subgroup analysis

A subgroup analysis was performed to investigate possible areas of heterogeneity by including studies that used different types of technological interventions. Group A (educational VR simulators) consists of studies in which a virtual reality system was employed to train skills such as bracket bonding and surgical planning. Group B (MR/AR and immersive assistive tech) consists of studies that used mixed reality systems, augmented reality to provide clinical support, or extremely immersive systems. The effect size in Group A is big and statistically significant (g = 1.40) and the effect size in Group B is big but not significant (g = 1.35, 95% CI: −0.23–2.93), which shows that there is a lot of uncertainty in the estimate in this subgroup. The overall impact of all the studies was big and significant (g = 1.37). The effect sizes and confidence intervals between the two subgroups significantly overlap visually, indicating that the type of technology is not a significant moderator of the overall effect.

**Figure 6:**
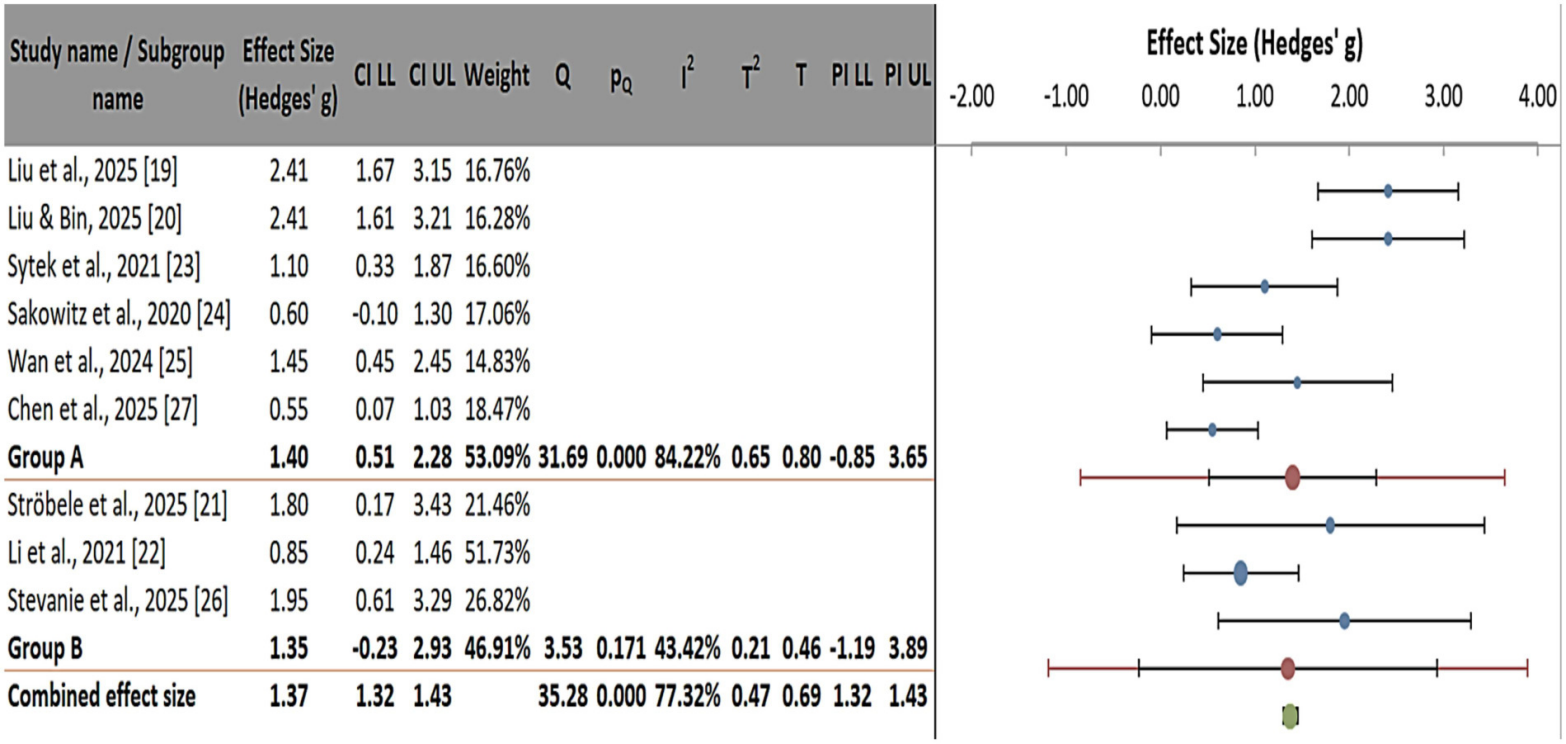
Subgroup analysis by type of technological intervention.

The test result for the subgroup difference supports the conclusion drawn from the visual inspection of the forest plot. According to the analysis of variance (ANOVA), the difference between the two groups is not statistically significant, with a *p*-value of 0.927. This indicates that the difference in the effect sizes between Groups A and B could be due to chance and not a real underlying difference in the efficacy of educational VR simulators compared to MR/AR and immersive assistive tech. The between-subgroups variance (Q^0^) is minimal (0.01) and the pseudo R 2 value is 0.13%, meaning that the effect of the type of technology is practically zero when considering the entire meta-analysis. The insignificant *p*-value of the total model (*p* = 0.613) also confirms that the subgroups are not significant in explaining the differences in effect size among the included studies (Figure 6 and Table 7).

**Table 7:** Test for subgroup differences (type of technology).

Meta-analysis model	Value		
Between-subgroup weighting	Random effects		
Within-subgroup weighting	Random effects (tau separate for subgroups)		
Confidence level	95%		
Combined effect size			
Correlation	1.37		
Standard error	0.02		
Confidence interval, lower limit	1.32		
Confidence interval, upper limit	1.43		
Prediction interval, lower limit	1.32		
Prediction interval, upper limit	1.43		
Number of included observations	228		
Number of included studies	9		
Number of subgroups	2		

Analysis of variance	Sum of squares (Q*)	df	*p*-Value
Between/model	0.01	1	0.927
Within/residual	6.29	7	0.506
Total	6.30	8	0.613
Pseudo R^2^	0.13%		

* Q* denotes the Q statistic/sum of squares used in the analysis of variance for subgroup differences.

**Figure 7:**
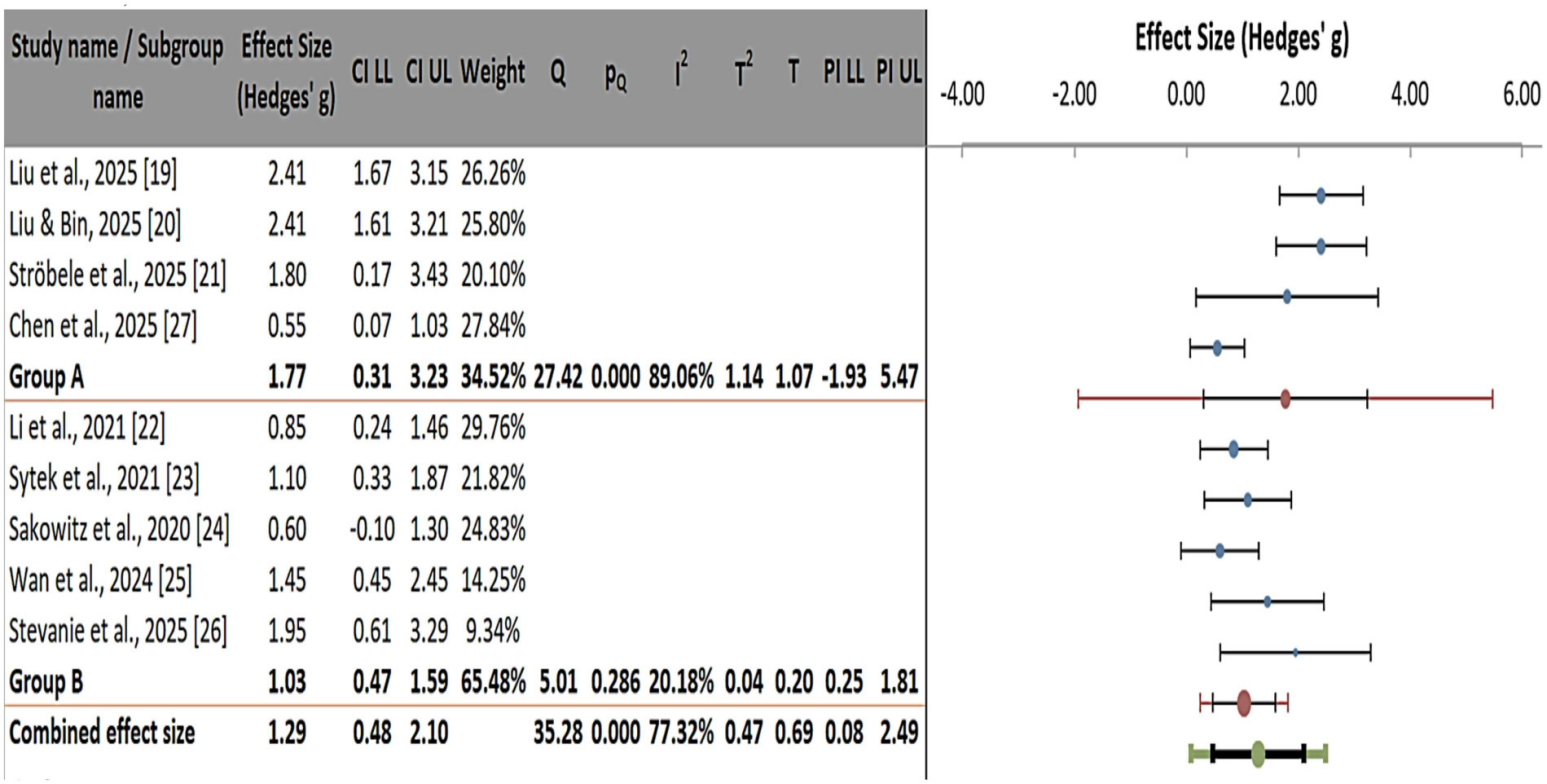
Forest plot of surgical vs. orthodontic/planning procedures: a subgroup analysis by clinical application.

Figure 7 is a forest plot of a subgroup analysis of the effect of the clinical implementation of a digital technology on the quantified effect size. The analysis shows that there is a clear divergence between the two procedure categories. The subgroup with the largest combined effect size (Hedges’ g = 1.77) is the bracket placement and bonding subgroup (Group A), although with very high and significant heterogeneity. This indicates that the technology is very effective for this type of orthodontic treatment when the average is considered, but the results are not consistent and may vary widely depending on the exact techniques and materials used or the skill of the operator. In sharp contrast, the effect in Group B, i.e., orthognathic surgical planning, training, and surgical adjuncts, is more moderate, reliable, and consistent (g = 1.03), with low non-significant heterogeneity. This suggests that the research studies demonstrate greater consistency and predictability in the technology’s benefit for these aspects of surgery. The high variance attributed to the clinical application/procedure type moderator justifies the subgroup analysis as it is an important factor that explains the high overall heterogeneity among the studies. Finally, the technology seems to provide a great, yet unstable, advantage in relation to orthodontic bonding procedures and provide more stable, moderate advantages in surgical planning and execution (Figure 7).

## Discussion

The results of this systematic review and meta-analysis indicate that the use of haptic and virtual reality technologies in orthodontic education and practice has a large and significant positive effect. The pooled effect size (g = 1.40) indicated these technologies significantly enhance performance indicators, i.e., bracket bonding accuracy, surgical planning skills, and skill acquisition rates, when compared to traditional approaches.

This is in line with an emerging body of literature in the medical and dental training field where simulation-based training has been proven over time to stimulate an improvement in psychomotor abilities and remediate competency gaps in novices due to secure, repeatable practice simulations (1, 3). For example, researchers in restorative dentistry established that haptic simulators enhanced cavity preparation skills. Moreover, our findings show that orthodontics-oriented skills, such as bracket placement, are equally enhanced when using immersive, force-feedback technology, with no clinical risk (19, 20, 27).

An important finding of this review is the high heterogeneity among the included studies (I^2^ = 77.32), which is unsurprising in an emerging field with substantial variation in technology platforms, learner populations, target procedures, outcome definitions, and instructional exposure. Beyond technology type alone, variability in session duration, frequency of practice, feedback architecture, and assessment method probably contributed to the effect size dispersion. A formal meta-regression exploring training duration was not feasible because only nine studies were available and the reporting of exposure variables was inconsistent across the studies. Nevertheless, the qualitative pattern suggests that the studies on bracket bonding showed wider variability because they differed more in simulator design, scoring systems, operator baseline experience, and repetition schedules than the studies that focused on orthognathic planning, which tended to use more standardized spatial-planning tasks.

In contrast, the orthognathic surgical planning, training, and surgical adjuncts subgroup showed a more predictable and moderate effect (g = 1.03). This is consistent with results in surgical specialties, where VR simulators have been shown to be especially useful for practicing complex spatial tasks and in procedural sequencing, as they present a structured and repeatable learning environment and standardize the acquisition of complex knowledge (23–25). The fact that these systems can generate three-dimensional and immersive anatomical reconstruction is a direct solution to the weaknesses of 2D planning algorithms, leading to increased confidence in diagnostics and accuracy of the planning (1, 23).

The practical implications of this article’s findings should be interpreted in proportion to the evidence. The strongest support at present is for educational use, as haptic VR simulators can enhance manual dexterity, spatial understanding, and procedural rehearsal in preclinical orthodontic curricula (2, 19). AR- or haptic-guided systems may also support operator accuracy during bracket placement, particularly for less experienced clinicians, but this should currently be viewed as assistance to the standard procedure rather than direct proof of improved patient outcomes. Future orthodontic applications may reasonably extend to clear-aligner attachment bonding, indirect bonding workflows, wire-bending rehearsal, and laboratory training for removable appliances; however, these areas remain underrepresented in the current evidence base and should be framed as future directions rather than established clinical benefits.

The risk-of-bias assessment further indicates the need for stronger study designs. A recurring challenge in educational technology trials is the practical difficulty of blinding participants to whether they used an immersive or haptic system, which may inflate self-reported outcomes such as confidence, satisfaction, or perceived realism. This limitation should be explicitly acknowledged when interpreting the present findings. Future trials should therefore prioritize objective performance outcomes, blinded external assessors for practical skill evaluations, standardized scoring rubrics, and longer follow-up to determine whether simulation-based gains persist and transfer to clinical settings (8, 9).

Overall, the evidence supports continued investigation and cautious implementation of haptic technology in orthodontic education and procedure planning. Its capacity to provide objective feedback and standardized simulation makes it a promising tool for improving the consistency of training and technical performance; however, extrapolation to broad patient-care transformation would be premature until direct clinical outcome studies become available (1, 3, 21).

### Limitations of the study

The principal limitation of this review is the substantial heterogeneity among the included studies, which could not be fully explained even after the subgroup analyses. Additional contributors likely include differences in simulator hardware, software algorithms, instructional duration, number of repetitions, assessor blinding, and baseline operator experience. The evidence base is also limited by small samples, moderate risk of bias in several studies, inconsistent outcome reporting, and the predominance of educational rather than patient-centered endpoints. These issues reduce certainty in the pooled estimate and limit strong conclusions about direct clinical effectiveness.

### Future directions

Future research should prioritize multicenter randomized controlled trials with larger samples, transparent reporting of training dose and feedback parameters, and follow-up periods sufficient to evaluate long-term retention and transfer to real clinical performance. Studies should include blinded external assessors wherever feasible and should expand beyond bracket bonding and orthognathic planning to other orthodontic procedures such as clear-aligner attachment placement, indirect bonding, wire bending, and removable-appliance fabrication. Most importantly, future investigations should distinguish educational efficacy from clinical effectiveness by incorporating patient-level and practice-level outcomes when such an evaluation is feasible.

## Conclusions

This systematic review and meta-analysis indicates that haptic and virtual reality technologies have a significant positive effect on orthodontic skill development and procedural planning. The strongest current evidence supports educational applications, particularly bracket-bonding training and orthognathic planning, while direct patient-level clinical benefits remain insufficiently demonstrated. Accordingly, these technologies should be viewed as promising adjuncts for orthodontic education and guided practice, pending stronger clinical outcome evidence.

## Data Availability

The original contributions presented in the study are included in the article/Supplementary Material; further inquiries can be directed to the corresponding authors.
